# Probabilistic graphical model for the evaluation of the emotional and dramatic personality disorders

**DOI:** 10.3389/fpsyg.2022.996609

**Published:** 2022-11-25

**Authors:** Jose D. García-Franco, Francisco J. Díez, Miguel Á. Carrasco

**Affiliations:** ^1^Department of Artificial Intelligence, Universidad Nacional de Educación a Distancia (UNED), Madrid, Spain; ^2^Department of Psychology of Personality, Evaluation and Treatment. Universidad Nacional de Educación a Distancia (UNED), Madrid, Spain

**Keywords:** personality disorder, probabilistic graphical model, Bayesian network, Delphi, artificial intelligence, knowledge engineering, decision support system

## Abstract

Personality disorders are psychological ailments with a major negative impact on patients, their families, and society in general, especially those of the dramatic and emotional type. Despite all the research, there is still no consensus on the best way to assess and treat them. Traditional assessment of personality disorders has focused on a limited number of psychological constructs or behaviors using structured interviews and questionnaires, without an integrated and holistic approach. We present a novel methodology for the study and assessment of personality disorders consisting in the development of a Bayesian network, whose parameters have been obtained by the Delphi method of consensus from a group of experts in the diagnosis and treatment of personality disorders. The result is a probabilistic graphical model that represents the psychological variables related to the personality disorders along with their relations and conditional probabilities, which allow identifying the symptoms with the highest diagnostic potential. This model can be used, among other applications, as a decision support system for the assessment and treatment of personality disorders of the dramatic or emotional cluster. In this paper, we discuss the need to validate this model in the clinical population along with its strengths and limitations.

## Introduction

We can define personality as the set of traits and qualities that shape a person’s way of being and differentiate him or her from others. According to DSM-5, personality disorders can be identified as an enduring pattern of inner experience and behavior that deviates markedly from the expectations of the individual’s culture. This pattern tends to be stable and of long duration; its onset can be traced back at least to adolescence or early adulthood and affect at least two areas of life (i.e., cognition, affectivity, interpersonal functioning, or impulse control) in an enduring, inflexible, pervasive way across a broad range of personal and social situations, which leads to clinically significant distress or impairment in social, occupational, or other important areas of functioning (American Psychiatric Association, 2013). While there exist uncountable different configurations that make the individual unique, some of them are more adaptive to the environment and society, while others can be considered dysfunctional, leading to significant psychological distress. Some maladaptive configurations are more prevalent than others and are often seen together; they are termed “personality disorders.”

The diagnosis and treatment of personality disorders have several challenges, such as the difficulty of diagnosing many of the maladaptive personality configurations under the current diagnostic approach, or the lack of consensus in the assessments due to evaluator biases. These difficulties are further analyzed in Section Evaluation of Personality Disorders.

The goal of this study is to develop a framework for the research and assessment of personality disorders in the emotional and dramatic cluster, which encompasses the antisocial (ATS), borderline (BDL), narcissistic (NAR), histrionic (HST), and passive-aggressive (PAG) disorders.

We apply artificial intelligence (AI) techniques to integrate different paradigms for the evaluation of personality disorders, which will provide clinicians with a more holistic and accurate tool that will allow them to assess relevant maladaptive psychological variables and psychological distress. This way, clinicians will have a more integral view of the relevant maladaptive psychological variables contributing to psychological distress, which could help reduce the clinical judgment biases derived from the differing backgrounds and profiles of the evaluators. Furthermore, it has been shown that diagnostic accuracy improves when the clinicians have the opportunity to reflect on their diagnosis assisted with the feedback and explanations offered by a decision support system (Oniśko, 2001).

The result of our work is a Bayesian network that models the most relevant psychological constructs related to the emotional and dramatic personality disorders. It contains a number of nodes representing those psychological constructs, a structure representing the relations of probabilistic dependence and independence among these constructs, and a set of conditional probabilities that allows us to draw inferences. These probabilities lead to some metrics, such as the likelihood ratio, which allows us to increase the diagnostic utility of screening and diagnostic tools.

This model allows us to infer the most probable diagnosis given a set of symptoms and find out the sources of psychological distress, which would make good therapeutic targets.

### The burden of personality disorders

Some studies indicate that the prevalence of personality disorder lies between 4.4 and 13.0% for the general population (Samuels et al., 2002; Coid, 2003; Lenzenweger et al., 2007; Huang et al., 2009), and can reach as high as 45% among psychiatric outpatients (Zimmerman et al., 2005). This variability can best be seen in Torgersen (2014) work.

Previous research suggests that, although some personality disorders may be considered ego-syntonic, the negative consequences for both the individual and his or her close relatives are significant, ranging from a decrease in both, quality of life (Torgersen, 2014), and life expectancy due to self-harming behaviors (Pompili et al., 2004; Krysinska et al., 2006; Zaheer et al., 2008), to problems with the law due to domestic violence (Whisman and Schonbrun, 2009) or criminal behavior (de Barros and de Pádua Serafim, 2008; Samuels, 2011). Personality disorders also impose a high cost on society as a whole due to the increased use of public health services (Chiesa et al., 2002) and absenteeism from work (Soeteman et al., 2008).

### Evaluation of personality disorders

Personality disorders are traditionally assessed by self-report questionnaires, rating scales, interviews, or projective techniques, with significant sources of variance (i.e., information, observation, interpretation, criterion). Many of these tools have not been constructed from an accurate psychometric perspective and have relied exclusively on clinical judgment, rather than an actuarial method, to arrive at a diagnosis (Westen and Shedler, 1999a). Even when some of the most popular and psychometrically well-founded tests (e.g., the Millon Clinical Multiaxial Inventory, MCMI; or the Minnesota Multiphasic Personality Inventory, MMPI) or structured interviews (e.g., Personality Disorder Interview–IV PDI–IV or the Structured Clinical Interview SCID–II) are used to make a diagnosis, they are often time-consuming and always have to be conducted by experienced or well-trained professionals. Moreover, these traditional procedures have focused mainly on the symptoms described in the DSM (Westen and Shedler, 1999a; Widiger and Lowe, 2011), which, in spite of being considered the “gold standard,” do not examine personality disorders from an integrated and holistic approach. As a result, the most frequently diagnosed personality disorder is the “Not Otherwise Specified” (Clark et al., 1997; Verheul and Widiger, 2004; Livesley, 2012) and 60% of patients in need of clinical psychotherapeutic attention due to a personality pathology are currently undiagnosable on DSM Axis II (Westen and Arkowitz-Westen, 1998).

Furthermore, the pressure imposed in successive revisions of the DSM to improve its internal and external validity, keeping at the same time a manageable number of symptoms (currently less than 10), helps explain the high comorbidity between personality disorders as well as the additional relations between symptoms and disorders beyond those described in the DSM (Westen and Shedler, 1999b). However, in real life, maladaptive personality is multifactorial and it is not conceivable that every patient fits neatly into a single personality disorder.

Due to these limitations, according to Westen and Shedler (1999a), most clinicians rely, primarily, on inferences drawn from the patient narrative of their lives and relations. This approach, while helping address the limitations previously discussed, is time-consuming and likely to induce a bias in the clinical judgment, which is known to reduce the diagnostic accuracy. Meehl (1954) proved that statistical judgment is up to 13% more accurate than clinical judgment (Ægisdóttir et al., 2006).

However, the biggest shortcoming and one of the main reasons that led scientists to push forward the research on personality disorders is the inadequate coverage of their different expressions (Widiger, 2007) and the lack of comprehensiveness (Westen and Shedler, 2000).

Given that the DSM has not yet provided an optimal solution for the evaluation of personality disorders, scientists have pursued other directions. Research has led to alternative frameworks that relate other psychological constructs to both general and individual personality disorders, such as the five-factor model (Lynam and Widiger, 2001; Widiger et al., 2002; Samuel and Widiger, 2004; Bagby et al., 2005), defense mechanisms (Berman and McCann, 1995; Cramer, 1999; Bowins, 2010), and Millon’s biosocial model (Piersma et al., 2002; Mullins-Sweatt and Widiger, 2007; Millon, 2011).

These alternative frameworks, which have the potential to discriminate those persons with an adaptive personality from those with a disordered personality, and also between different personality disorders, are not generally used, *per se*, for the diagnosis of personality disorders, even though these frameworks are supported by empirical research or by a solid theoretical basis.

Most assessment tools are based on the DSM criteria (Widiger and Lowe, 2011), so these limitations apply, to more or less an extent, to the usual evaluation questionnaires used nowadays by clinical psychologists; hence, the need to incorporate these alternative frameworks into the evaluation of personality disorders. The advantages of a unified framework that increases coverage of symptoms by including all the psychological constructs related to personality disorders justify our research, as nowadays the treatment of personality disorders is individualized, aiming at the person’s symptoms rather than at the disorder itself (Millon and Grossman, 2007; Millon and Grossman, 2007a,b). Furthermore, a more comprehensive measurement tool could allow us to reduce biases, both those induced by the person being evaluated, since we would have more information on which to make a decision, as well as those of the evaluator since it could enhance his/her clinical judgment with a statistical/probabilistic tool.

### Decision support systems in psychology

One of the main applications of AI is the development of expert systems which are software programs able to mimic the human decision process (Saibene et al., 2021). Many expert systems have been built for different medical domains, but very few for psychology. Saibene et al. (2021), in a five-year review of the literature, identified 43 studies regarding the application of expert systems in healthcare; only 2 were related to psychology, and none of them to personality or its disorders although Luxton (2014) had identified several areas of psychology where the use of AI technology could make a difference.

From 2015 onward there has been, according to Graham et al. (2019), a steep increase in the number of publications about AI for mental health. However, our database search (Scopus, Web of Science, Science Direct, PubMed, IEEE Xplore) with the terms “expert system,” “decision support system,” or “artificial intelligence” on the one hand, and “personality disorders” or any of the individual disorders on the other, only returned tangential research (Singh et al., 2020; Ellouze et al., 2021; Khazbak et al., 2021), proposals (Tuena et al., 2020; Sulistiani et al., 2021; Szalai, 2021), or proofs of concept (Nunes et al., 2009; Casado-Lumbreras et al., 2012; Randa and Permanasari, 2014; Laijawala et al., 2020).

We conjecture that this scarcity of decision support systems in the field of personality disorders may be, in part, because psychological diagnosis is based on phenomenology. Thus, it can be highly subjective as it depends on the experiences of a person with psychological problems. Conversely, medical diagnosis is often helped by laboratory results and other objective quantitative measures, in addition to clinical signs (Fernando et al., 2011). However, an application of Bayesian methods that is gaining importance nowadays is the analysis of networks in which, through a directed acyclic graph and machine learning techniques, an attempt is made to determine the causal relations between the nodes in the network (Briganti et al., 2020; Černis et al., 2021).

Furthermore, there are two trends to build expert systems. One consists in eliciting and encoding the knowledge of human experts; the other, in applying machine learning algorithms to a large dataset (Constantinou et al., 2016). The latter has the problem that curated medical data regarding psychiatric disorders is generally unavailable (Suhasini et al., 2011). In the case of knowledge-based systems, the problem is that the causal mechanism that drives the relations among variables is either poorly understood or mediated by a large number of hidden variables, which makes it very difficult to elicit expert knowledge; additionally, obtaining the numerical parameters for these systems is even more difficult. Moreover, many AI classification techniques, such as neural networks and support vector machines (SVMs) only work with large data sets and not with expert knowledge.

To achieve the proposed goals, we present in Section 2 the methodology used, and in Section 3 the structure of the resulting model, the raw probabilities obtained, and the likelihood ratios for the symptoms of personality disorders. We conclude the presentation with a discussion of the model and its applications in clinical and research settings (Section 4).

## Materials and methods

### Participants

We recruited two groups of psychologists with academic and/or clinical expertise in the diagnosis and treatment of personality disorders.

The first group (n=5), which has several years of clinical experience (M=12;SD=7), was tasked with validating the psychological variables, identified through a literature search, and the structure of the model.

The second group (n=7), also having several years of experience (M=20;SD=15), was responsible for obtaining the conditional probability tables used as parameters in the model.

### Instruments

For the development of the model, a set of questionnaires was used to define the structure of the model and another set to obtain the conditional probabilities. These questionnaires were custom-made and tailored to obtain the causal links among nodes and the probabilities of the symptoms conditioned on the disorders.

All the questionnaires were completed using forms embedded within PDF files, which could be received, answered, and sent back electronically, thus facilitating the participants’ engagement.

For the identification of the causal relations between personality disorders and symptoms, the experts were provided with a questionnaire with several tables, one for each psychological framework. For each table, every row corresponds to one of the symptoms, and every column to one of the five personality disorders. The questionnaire consisted of checkboxes (one per cell on each table), which allowed entering a yes/no answer indicating whether the symptom is related to the personality disorder.

Symptoms and dependency links were previously established through a literature review and the study of different psychological measurement instruments for personality disorders. The relations cited as relevant in the literature had previously been checked. Participants were instructed to unmark the checkbox should they consider that a relationship is not sufficiently relevant (if it was previously checked) or leave it blank (if it was not). Similarly, if the experts considered that a symptom was related to a particular personality disorder, they were instructed to mark the checkbox if it was not already marked, or leave it checked if it already was, thus validating the previous literature search.

To standardize the interpretation of symptoms, we briefly described them in the questionnaire. Furthermore, at the end of the form, there was a free-text field so that the experts could add any missing psychological constructs and their relations with the disorders.

To obtain the parameters of the model, the second group of experts was given a set of questionnaires classified by personality disorder.

Again, the rows corresponded to the symptoms but, in this case, through the columns, we sought the probability that the symptom defined in the row would be present when: (*a*) the personality disorder was also present, (*b*) when the personality disorder was absent (control group) and (*c*) the probability that the symptom may cause significant psychological distress.

The scale for data input consisted of a rating scale from 0 to 100. This scale was conceptually divided into four intervals, which were assigned four probability categories: 0–25 “not probable,” 25–50 “improbable,” 50–75 “probable,” and 75–100 “very probable.” A graph depicting this division was printed on the header of each page and served as a guide for the psychologist, who is usually more familiar with Likert scales, to elicit the probabilities. The answers were recorded on numerical text fields in each cell, which allowed entering a value between 0 and 100.

Following the Delphi method, the first questionnaire was common to all the participants. This form included, as items, all the parameters that we would need for the construction of the model.

In the next round, a personalized form was used for each participant. For those items in which there was no consensus, defined as those answers that were more than one standard deviation away from the mean, his/her previous response, as well as aggregated data about the responses of other experts, were included. The participant had the chance to modify the previous answer or to keep it. For those items for which there was consensus, it was not allowed to modify the previous answer.

### Procedure

The participants in this research received by e-mail a letter of introduction and an invitation to participate in the project. No expert ever knew the identity of the others. All questionnaires included instructions for their correct completion and a demographic data form.

Regarding the structure of the model, the dependency relations finally included were those for which there was consensus (simple majority) among the first group of experts. We anticipated that those relations for which there was no clear consensus would not be sufficiently relevant to significantly affect the accuracy of the model, given that probabilities would be assigned based on the strength of that relation.

The probabilities for the model were obtained using the Delphi method, with at least two rounds. After the first round, the experts were provided with aggregated data (mean and standard deviation) of the answers given in the previous round by all the participants. Each expert could keep his/her previous response or modify it. The process ended when a consensus had been reached or when no further progress was obtained after successive rounds.

According to Hsu and Sandford (2007), the key factor for the success of the Delphi technique is the choice of experts. The number of participants should be enough to obtain a representative sample of expert opinions (Latif et al., 2016), but an excessive number would slow down the process without a substantial improvement in accuracy (Hsu and Sandford, 2007).

In a systematic review of consensus-building methods, Waggoner et al. (2016) suggest having 6 to 11 participants. As previously mentioned, we involved 7 experts in this phase.

The number of rounds required in the methodology is not established. Waggoner et al. (2016) propose a minimum of two rounds, which is the minimum required to obtain at least one feedback from their colleagues. However, although no maximum number of rounds is established, other authors, like Hasson et al. (2000) and Woudenberg (1991), argue that two rounds are usually sufficient, as this is when maximum accuracy is reached. We have used two rounds in this research since, after analyzing the results of the second one, we saw an obvious risk of a regression to the mean, thus reducing the diversity of responses.

Although the use of the Delphi methodology to obtain conditional probability tables seems promising, we have only found two studies using it (Chen and Huang, 2018; Wu et al., 2018). However, the details of the implementation of the method are not described in those papers, so we have relied on a general approach (Hasson et al., 2000; Waggoner et al., 2016) and adapted it to our research.

The value finally selected for each probability was the average of the responses in the last round.

### Development of the probabilistic graphical model

A probabilistic graphical model (PGM) is an encoded probability distribution in which the variables are represented as nodes and the dependence relations as edges between nodes.

A Bayesian network (BN) is a type of PGM consisting of an acyclic directed graph and a conditional probability table for each node given its parents,


$$P\left(X_{i}\;|\;\mathrm{pa}\left(X_{i}\right)\right).$$

The joint probability implicitly represented by a BN is:


$$P\left(X_{1}{\mathrm{,X}}_{2}\ldots X_{n}\right)=\underset{i}{\prod }\;P\left(X_{i}\;|\;\mathrm{pa}\left(X_{i}\right)\right),$$

where $\mathrm{pa}\left(X_{i}\right)$ is the set of parents of node $X_{i}$ in the graph.

A *finding* determines with certainty the state of a variable; for example, the value “true” or “high.” The set of all the findings available at a point in time is called *evidence*.

Probabilistic reasoning consists in calculating the posterior probabilities of variables of interest that are not in the evidence.

One advantage of BN is the ease of integrating statistical data with expert knowledge. Another one is the possibility of working with missing data. Furthermore, BN have good accuracy even with small data sets with the use of canonical models (Oniśko et al., 2001) or when probabilities are not overly precise (Uusitalo, 2007).

The most common sources of information to build Bayesian networks are statistical data, scientific literature, and human experts (Druzdzel and van der Gaag, 2000). In this research, we have combined a search of the scientific literature and knowledge elicitation from human experts.

The construction of a probabilistic graphical model for a given domain has three phases; identifying the variables, defining the structure of the model and obtaining the conditional probabilities (Druzdzel and van der Gaag, 2000). We have carried out them using the graphical user interface of OpenMarkov, an open-source tool (Arias et al., 2011) and then exported the model to the academic version of GeNIE (Druzdzel, 1999) to take advantage of its graphing capabilities.

We should note that, although OpenMarkov is very useful for building Bayesian networks, we can benefit from customized software development that acts as an interface between the user and the model. Such an interface, which we developed in conjunction with the Bayesian network throughout this research, improves the usability of the system and allows a clinician to interact with the model without the need to know about Bayesian networks or their building tools.

#### Identification of the relevant variables, the type of variable (continuous or discrete) and the number of different states

The variables included in the model should cover as broadly as possible the psychological spectrum related to the personality disorders that we want to assess, but without including duplicated or highly correlated variables.

These psychological constructs should be easily measurable and, if possible, familiar to the clinical psychologists who will make use of the decision support system. Therefore, the selection of those variables was performed using the “snowball” method of literature review, taking as starting points papers about commonly used questionnaires for the diagnosis of personality disorders.

Included in the model as nodes are all the symptoms of the classical DSM diagnostic method. None of the specific constructs from the alternative dimensional diagnostic method published in the latest version of the DSM were considered due to the small amount of research on this new model and the absence of some personality disorders (i.e., narcissistic, histrionic and passive-aggressive personality disorders). However, since this dimensional model is an adaptation of the older five-factor model, its exclusion will not have a negative impact because the same psychological constructs are covered by the five-factor model which, additionally, has been extensively used as a personality measurement instrument and in relation to personality disorders (Costa and Widiger, 2002; Widiger and Costa, 2013).

Regarding the five-factor model, we have included in our model all the traits from the domains of neuroticism, extraversion, and agreeableness and all the traits of openness and conscientiousness, except the traits of aesthetics, ideas, values, and achievement-striving, which are the ones that, according to the majority of the studies reviewed (Lynam and Widiger, 2001; Widiger et al., 2002; Samuel and Widiger, 2004; Bagby et al., 2005) did not have a strong relation with personality disorders of the dramatic or emotional type.

The psychological constructs of the DSM-5 new diagnostic method that capture the severity of the personality disorder (Hutsebaut et al., 2016) has been included. These variables, namely identity, empathy, intimacy, and self-direction, correspond to the general factors common to all the personality disorders and match the four scales of the level of personality functioning (LPFS; Hopwood et al., 2018).

In addition to the variables related to the diagnosis of personality and its disorders, other variables that facilitate the differential diagnosis have been included in the model, such as defense mechanisms (acting out, idealization, denial, dissociation, devaluation, projection, projective identification, splitting, displacement, and passive aggression; American Psychiatric Association, 2000) and the six polarities (pleasure, pain, active, passive, self, other) from the Millon’s biosocial theory related to the maladaptive configurations of the individual’s styles of adaptation to the environment (Millon, 2011).

Along with the variables we have just described, which correspond to the symptoms, we have also included in the model five nodes corresponding to the personality disorders, as well as other nodes (14 in total) that we use to measure the psychological distress that cluster of symptoms may produce in the patient.

Although the measurements for the psychological variables and even the personality disorders are continuous in nature, we have discretized all the variables. This is a common approach, as there are no efficient algorithms to deal with Bayesian networks that include continuous variables, either for inference or learning, even for very simple models.

Furthermore, given that the computational complexity increases very fast with the number of states, we have only used binary variables (yes/no, present/absent) for the DSM framework and for the defense mechanisms. The nodes representing the personality disorders themselves and the psychological distress have been also modeled as binary variables.

Variables from the level of personal functioning, the five-factor, and the biosocial models have been discretized into three states: low, medium, and high. However, for the five-factor and the biosocial models, the medium state not only indicates a point between the other extreme values, but also it implies that the score obtained is not significant and that it falls within the population mean.

#### Identifying and representing the causal relations

We have modeled the network assuming that personality disorders cause the symptoms. This way we limit the number of ancestor nodes and reduce the overall complexity of the model. Therefore, a node will only have as many ancestors as the number of personality disorders that may cause it.

**Figure 1:**
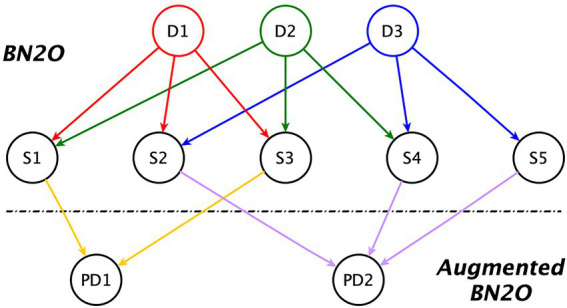
Augmented BN2O model. D_x_ = Disorder; S_y_ = Symptom; PD_z_ = Psychological distress.

An overview of the model structure is presented in Figure 1.

The first two levels of that figure correspond to a BN2O model, which is widely used in medical expert systems (Heckerman, 1990). It consists of an upper level whose nodes represent possible diagnostics, and a lower level (the middle level in our figure), containing the symptoms, observations, medical tests, etc.

The third level in the figure is an extension to the model, first introduced in this research. When introducing evidence about the symptoms, those that are absent may cancel the impact of those that are present, leading to a false-negative diagnosis. The third level in the model alleviates the problem by allowing us to detect clusters of maladaptive symptoms even when the diagnosis is negative. These nodes, which represent the psychological distress in the individual, are also used to perform a sensitivity analysis and to indicate the best therapeutic targets for treatment.

We can observe in the figure that there are no dependency links between diagnoses, which would indicate comorbidity, or between symptoms, which would indicate some kind of correlation among them. The absence of relations between symptoms is deliberate, motivated by the need to reduce the complexity of the model. On the one hand, we have avoided introducing highly correlated symptoms, as it would be redundant, and, on the other, weak dependencies are usually removed given that they do not significantly change the results in classification tasks (Kjærulff, 1994). Furthermore, the inclusion of these relations would not affect the diagnosis given that, when we make a node deterministic by introducing a finding, its state is not affected by the probabilities given its ancestor nodes. As for comorbidity between diagnoses, while it is documented between personality disorders, we model this comorbidity through the common symptoms that these disorders have; hence, the lack of direct links among disorders.

The initial list of dependency links between symptoms and personality disorders for the probabilistic graphical model was obtained from the same literature review used to identify the relevant psychological constructs, and then peer-reviewed by the team of experts, as explained above, using the questionnaire designed for this purpose.

#### Obtaining the conditional probabilities

Probabilistic graphical models allow for the combination of experimental data with expert knowledge. Since a sufficient amount of suitable data is rarely available in the field of mental health (Suhasini et al., 2011), the probabilities associated with the nodes were elicited from a group of experts. However, a person’s experience may be biased by his/her professional experience; we overcome this drawback by using the Delphi methodology for obtaining a consensus, as explained in Section 2.3.

One of the advantages of this method, in addition to the elimination of outlier answers, is that it encourages the participants to reflect on their answers, thus reducing idiosyncratic biases or a tendency to answer too quickly due to fatigue and the large number of items.

The results obtained through the questionnaires are the raw probabilities that indicate the chance that the symptom is present when a single personality disorder is also present (or absent). To obtain the conditional probability tables for the model, it is necessary to first carry out a transformation, due to the difficulty of eliciting from the experts the probabilities of the symptoms when we have to take into account the joint presence or absence of several personality disorders simultaneously.

Moreover, the presence of a large number of ancestor nodes causes an exponential increase in computational complexity (an instance of “the curse of dimensionality”), which we have solved by using canonical models (Diez and Druzdzel, 2006) and taking advantage of the “independence of causal influence” property. This property assumes that the impact of a single cause on the effect does not depend on other causes that may exist, their order, or their interaction (Heckerman and Breese, 1994). Furthermore, canonical models allow complexity to grow linearly with the number of ancestor nodes. So, despite obtaining an approximation to the true values, we actually may gain accuracy by simplifying the elicitation of expert knowledge.

Regarding our model, for two-state variables, we used a “leaky OR” model, and for those three-state variables whose “neutral” state—understood as the absence of disorder or anomaly—is the lowest, we used a “leaky MAX.” For an in-depth review of these and other canonical models, see (Diez and Druzdzel, 2006).

However, the above-mentioned canonical models are not adequate for modeling all of the three-state nodes because: (*a*) some nodes behave as inhibitors themselves, that is, they reduce the probability that the symptom is present when a given disorder is also present; and (*b*) for these three-state variables, the default state is not its lowest.

To deal with these variables, we have developed a novel canonical model that allows us to work with multi-state variables without the limitations described above. Its rationale is that there are causes that count as evidence in favor of a given effect. The more evidence we have, either because given the cause the effect is very likely, or because there are several causes supporting the effect, the greater the probability that said effect is present. Conversely, the more evidence against the effect, the less likely it is to be present. We assume that, as in clinical diagnosis by professionals, the probability of the effect (a symptom) depends on the weighting of the evidence for and against, taking into account that not all findings have the same diagnostic potential.

The raw probabilities we obtained using the Delphi method, besides being necessary for generating the conditional probability tables for the model, allow us, for each symptom, to calculate the likelihood ratio with respect to each personality disorder, which is a widely used metric in clinical settings for measuring diagnostic strength.

The positive likelihood ratio for a test result indicates the magnitude of the increase in the probability of a given disorder when the test is positive. Conversely, the negative likelihood ratio for a test result indicates the decreased likelihood of a given disorder when the test is negative (Hayden and Brown, 1999; Grimes and Schulz, 2005).

By identifying symptoms with a higher positive likelihood ratio, we can develop a reduced measurement instrument to confirm the presence of personality disorders of the dramatic and emotional type in a clinical setting. Conversely, by identifying symptoms with a lower negative likelihood ratio we can design a screening instrument to rule out the presence of those personality disorders in the general population.

## Results

### Raw probabilities obtained with the Delphi methodology

The results presented in the following tables are the probabilities that each symptom is present when the personality disorder (ATS, BDL, NAR, HST, or PAG) is also present, the probability that the symptom is present in the absence of any personality disorder (Norm.) and the psychological distress the symptom may provoke (PD).

For ease of reading, the results have been split into different tables and classified by diagnostic framework: DSM (Table 1), defense mechanism (Table 2), level of personality functioning (Table 3), five-factor model (Table 4), and Millon’s biosocial model framework (Table 5). The prevalence of personality disorders is shown in Table 6 for both the clinical and the general population.

**Table 1:** Probabilities (%) of DSM symptoms for cluster-B personality disorders.

DSM symptom	Personality disorders					Norm.	PD
	ATS	BDL	NAR	HST	PAG		
DSM-ATS-01	76.4	—	—	—	—	11.4	46.4
DSM-ATS-02	81.4	—	—	—	—	27.9	28.6
DSM-ATS-03	64.3	75.0	—	—	—	36.4	52.1
DSM-ATS-04	77.1	70.7	—	—	—	35.0	60.7
DSM-ATS-05	65.7	66.4	—	—	—	25.7	41.4
DSM-ATS-06	81.4	—	—	—	—	22.9	36.4
DSM-ATS-07	80.7	—	73.6	—	—	11.4	27.1
DSM-BDL-01	—	81.4	—	64.3	—	26.4	69.3
DSM-BDL-02	—	86.4	—	65.0	—	17.9	67.1
DSM-BDL-03	—	88.6	—	—	—	11.4	76.4
DSM-BDL-04	—	85.7	—	—	—	17.1	78.6
DSM-BDL-05	—	76.4	—	—	—	15.7	78.6
DSM-BDL-06	—	85.7	—	72.1	—	17.9	79.3
DSM-BDL-07	—	82.1	—	—	—	16.4	79.3
DSM-BDL-08	75.7	80.7	—	—	—	22.9	72.9
DSM-BDL-09	—	63.6	—	40.7	—	10.0	75.7
DSM-NAR-01	—	—	85.7	—	—	23.6	14.3
DSM-NAR-02	—	—	85.7	—	—	22.9	16.4
DSM-NAR-03	—	—	91.4	—	—	25.0	19.3
DSM-NAR-04	—	—	90.0	80.0	—	22.1	26.4
DSM-NAR-05	—	—	84.3	—	—	23.6	14.3
DSM-NAR-06	—	—	85.7	—	—	29.3	25.0
DSM-NAR-07	79.3	—	77.1	—	—	16.4	22.1
DSM-NAR-08	—	—	77.1	—	77.9	32.1	23.6
DSM-NAR-09	—	—	86.4	—	—	24.3	19.3
DSM-HST-01	—	—	—	87.9	—	16.4	48.6
DSM-HST-02	—	—	—	81.4	—	19.3	45.0
DSM-HST-03	—	—	—	78.6	—	21.4	55.7
DSM-HST-04	—	—	—	81.4	—	22.1	35.0
DSM-HST-05	—	—	—	77.9	—	22.1	27.1
DSM-HST-06	—	—	—	87.9	—	15.7	42.1
DSM-HST-07	—	63.6	—	82.1	—	25.0	35.7
DSM-HST-08	—	62.1	—	80.7	—	17.1	44.3
DSM-PAG-01	67.1	—	—	—	82.9	22.1	57.1
DSM-PAG-02	—	—	—	61.4	77.9	17.1	57.9
DSM-PAG-03	72.9	—	—	—	77.1	22.1	67.9
DSM-PAG-04	75.0	—	—	—	76.4	22.9	57.9
DSM-PAG-05	—	—	65.0	—	74.3	22.9	52.9
DSM-PAG-06	—	—	—	—	76.4	24.3	57.9
DSM-PAG-07	—	—	—	—	86.4	19.3	64.3

ATS = antisocial; BDL = borderline; NAR = narcissistic; HST = histrionic; PAG = passive-aggressive; Norm. = normative (no personality disorder); PD = psychological distress.

**Table 2:** Probabilities (%) of defense mechanisms for cluster-B personality disorders.

Defense mechanism	Personality disorders					Norm.	PD
	ATS	BDL	NAR	HST	PAG		
Acting Out	85.7	84.3	—	70.0	—	27.9	60.0
Idealization	—	67.1	—	—	—	27.1	44.3
Denial	75.7	78.6	80.0	77.1	—	38.6	28.6
Dissociation	47.1	—	55.0	72.1	—	15.0	55.0
Devaluation	—	85.0	44.3	—	—	17.9	69.3
Projection	76.4	—	70.0	—	—	42.1	34.3
Projective identification	—	—	—	—	77.9	21.4	62.9
Splitting	—	87.9	—	72.1	—	22.9	64.3
Displacement	—	—	—	—	70.0	24.3	54.3
Passive aggression	—	71.4	—	58.6	88.6	24.3	48.6

ATS = antisocial; BDL = borderline; NAR = narcissistic; HST = histrionic; PAG = passive-aggressive; Norm. = normative (no personality disorder); PD = psychological distress.

**Table 3:** Probabilities (%) of level of personality functioning (LPF) scales for cluster-B personality disorders.

LPF scale	Personality disorders					Norm.	PD
	ATS	BDL	NAR	HST	PAG		
Identity	69.3	87.9	65.7	77.9	67.1	15.0	57.9
Self-direction	62.1	80.0	51.4	65.0	70.0	22.1	49.3
Empathy	85.0	75.7	65.0	70.0	78.6	15.0	27.1
Intimacy	80.0	79.3	43.6	75.7	69.3	12.9	45.7

ATS = antisocial; BDL = borderline; NAR = narcissistic; HST = histrionic; PAG = passive-aggressive; Norm. = normative (no personality disorder); PD = psychological distress.

**Table 4:** Probabilities (%) of five-factor model (FFM) traits for cluster-B personality disorders.

FFM trait	Personality disorders					Norm.	PD
	ATS	BDL	NAR	HST	PAG		
Anxiety	↓ 57.9	↑ 77.9	—	—	—	44.3	70.7
Angry hostility	↑ 77.1	↑ 80.7	↑ 62.9	—	↑ 77.1	35.7	52.1
Depression	—	↑ 77.1	—	↑ 47.9	—	46.4	77.9
Self-consciousness	↓ 67.9	—	—	—	—	34.3	71.4
Impulsiveness	↑ 83.6	↑ 83.6	—	—	—	37.1	55.7
Vulnerability	—	↑ 80.0	—	↑ 68.6	—	32.9	75.0
Warmth	↓ 63.6	↓ 48.6	↓ 63.6	—	—	32.9	34.3
Gregariousness	↓ 54.3	↓ 38.6	—	↑ 75.0	—	24.3	38.6
Assertiveness	—	—	↑ 62.9	—	↓ 77.1	33.6	61.4
Activity	—	—	—	↑ 57.9	—	47.9	25.7
Excitement seeking	↑ 65.0	—	↑ 49.3	↑ 65.7	—	41.4	30.0
Positive emotions	—	—	—	↑ 54.3	—	27.9	70.7
Fantasy	—	↑ 60.0	↑ 79.3	↑ 77.9	—	35.0	N/A
Feelings	—	—	—	↑ 57.9	—	25.7	N/A
Actions	—	↑ 43.6	—	↑ 65.7	—	33.6	N/A
Trust	↓ 75.0	↓ 65.0	↓ 56.4	↑ 59.3	↓ 73.6	38.6	45.7
Straightforwardness	↓ 84.3	↓ 62.1	↓ 73.6	—	↓ 75.0	35.7	24.3
Altruism	↓ 86.4	—	↓ 76.4	—	—	33.6	18.6
Compliance	↓ 86.4	↓ 70.0	↓ 75.7	—	↓ 75.7	27.1	46.4
Modesty	↓ 65.0	—	↓ 87.1	—	—	38.6	24.3
Tender-mindedness	↓ 80.7	—	↓ 75.0	—	—	24.3	17.1
Competence	—	↓ 75.7	↑ 76.4	—	↓ 70.7	25.0	69.3
Order	—	↓ 54.3	—	—	—	36.4	36.4
Dutifulness	↓ 80.7	—	—	—	↓ 70.0	32.1	28.6
Self-discipline	↓ 68.6	—	—	—	↓ 64.3	40.0	45.7
Deliberation	↓ 74.3	↓ 82.1	—	↓ 70.0	—	32.9	45.7

ATS = antisocial; BDL = borderline; NAR = narcissistic; HST = histrionic; PAG = passive-aggressive; Norm. = normative (no personality disorder); PD = psychological distress; N/A = not applicable.

Upward arrow = direct relation between symptom and disorder; downward arrow = inverse relation.

**Table 5:** Probabilities (%) of polarities for cluster-B personality disorders.

Polarity	Personality disorders					Norm.	PD
	ATS	BDL	NAR	HST	PAG		
Pleasure	—	↓ 72.9%	↑ 77.1%	↑ 58.6%	↓ 57.1%	↑ 40.0% / ↓ 22.5%	N/A
Pain	—	↑ 67.9%	—	↓ 44.3%	↑ 72.1%	↑ 30.0% / ↓ 20.0%	N/A
Active	—	—	↑ 74.3%	↑ 55.0%	—	↑ 47.5%	N/A
Passive	—	↑ 56.4%	—	↓ 63.6%	↑ 59.3%	↑ 25.0% / ↓ 22.5%	N/A
Self	↑ 82.1%	—	↑ 85.7%	↓ 41.4%	—	↑ 30.0% / ↓ 15.0%	N/A
Other	—	—	—	↑ 20.7%	—	↑ 20.0%	N/A

ATS = antisocial; BDL = borderline; NAR = narcissistic; HST = histrionic; PAG = passive-aggressive; Norm. = normative (no personality disorder); PD = psychological distress; N/A = not applicable.

Upward arrow = direct relation between symptom and disorder; downward arrow = inverse relation.

**Table 6:** Prevalence (%) of dramatic and emotional personality disorders and psychological distress.

Personality disorder	Prevalence		PD
	Clinical population	General population	
Antisocial	12.4	2.4	70.0
Borderline	19.3	3.5	87.1
Narcissistic	11.9	4.3	61.4
Histrionic	13.3	3.6	72.9
Passive-aggressive	9.1	3.0	62.1

PD = psychological distress.

Most of the symptoms described here are maladaptive, i.e., they have a positive correlation with the personality disorder (which is also maladaptive). However, for the five-factor model (Table 4) and Millon’s biosocial model (Table 5), the presence of a symptom may imply an increase in probabilities with one disorder but a decrease in probabilities with another disorder. A direct relation is represented by an upward pointing arrow and an inverse relation by a downward arrow.

The results obtained correspond to the average of the probabilities provided by the experts in the final round of the Delphi method. However, it is interesting to mention that the consensus degree of the experts in the first round was, on average, similar for all the personality disorders (66.43%±12.10%).

In the second round, the experts modified a considerable number of responses that fell outside the range of consensus by the experts (79.63%±25.80%), but the consensus degree raised only slightly (72.21%±10.76%). The average probability for the presence of a symptom in the presence of the corresponding personality disorders was 71.92%±11.08%. Alternatively, the average probability of the presence of a symptom in the absence of any personality disorder was 25.05%±9.00%.

As for the clinically significant psychological distress that the symptoms described in the model are capable of producing, we obtained a mean probability of 47.63%±19.03%.

### Probabilistic graphical model

Given the structure of the model validated by the first group of experts and the raw probabilities obtained from the second group of experts, we built the Bayesian network.

#### Nodes of the model

The nodes of the model correspond to all the psychological variables and symptoms listed in the first column of the aforementioned tables. Additionally, it should be added the five nodes corresponding to the five personality disorders we are evaluating and the 14 nodes related to the psychological distress caused by each symptom grouping.

These 14 nodes are distributed as follows: one for each personality disorder in the DSM model (5 in total), 4 for each domain in the FFM model (all except for openness), 3 for the personal functioning scale, one for the defense mechanisms, and a final one that measures the general psychological distress caused by personality disorders.

#### Structure of the model

The structure of the model can be determined based on the tables themselves, taking into account that the existence of a probability between symptom and disorder, as seen in the aforementioned tables, implies an arc in the graphical representation.

Furthermore, each of the 14 nodes that account for the psychological distress is linked with the nodes that represent the symptoms or the personality disorders causing that psychological distress.

#### Parameters of the model

For the nodes corresponding to the psychological variables listed under the DSM (Table 1) and the defense mechanisms (Table 2) frameworks, the conditional probabilities were obtained by using the probabilities directly if the node has only one ancestor node, or with the help of a canonical model “leaky OR” otherwise (Diez and Druzdzel, 2006).

For the level of personality functioning paradigm (Table 3), the conditional probability tables are obtained using the canonical “leaky MAX” model (Diez and Druzdzel, 2006).

For the five-factor model (Table 4) and Millon’s biosocial model framework (Table 5), we have used a logistic-Gaussian canonical model specifically designed for this research, which allows us to overcome some of the limitations of other canonical models and to take into account the differing prevalence of each symptom, trait, or scale in the population.

For those nodes that have no ancestors, i.e., for each of the five personality disorders, the conditional probability coincides with the prevalence (obtained as well by the Delphi method), which is shown in Table 6 for both the clinical and the general population.

**Figure 2:**
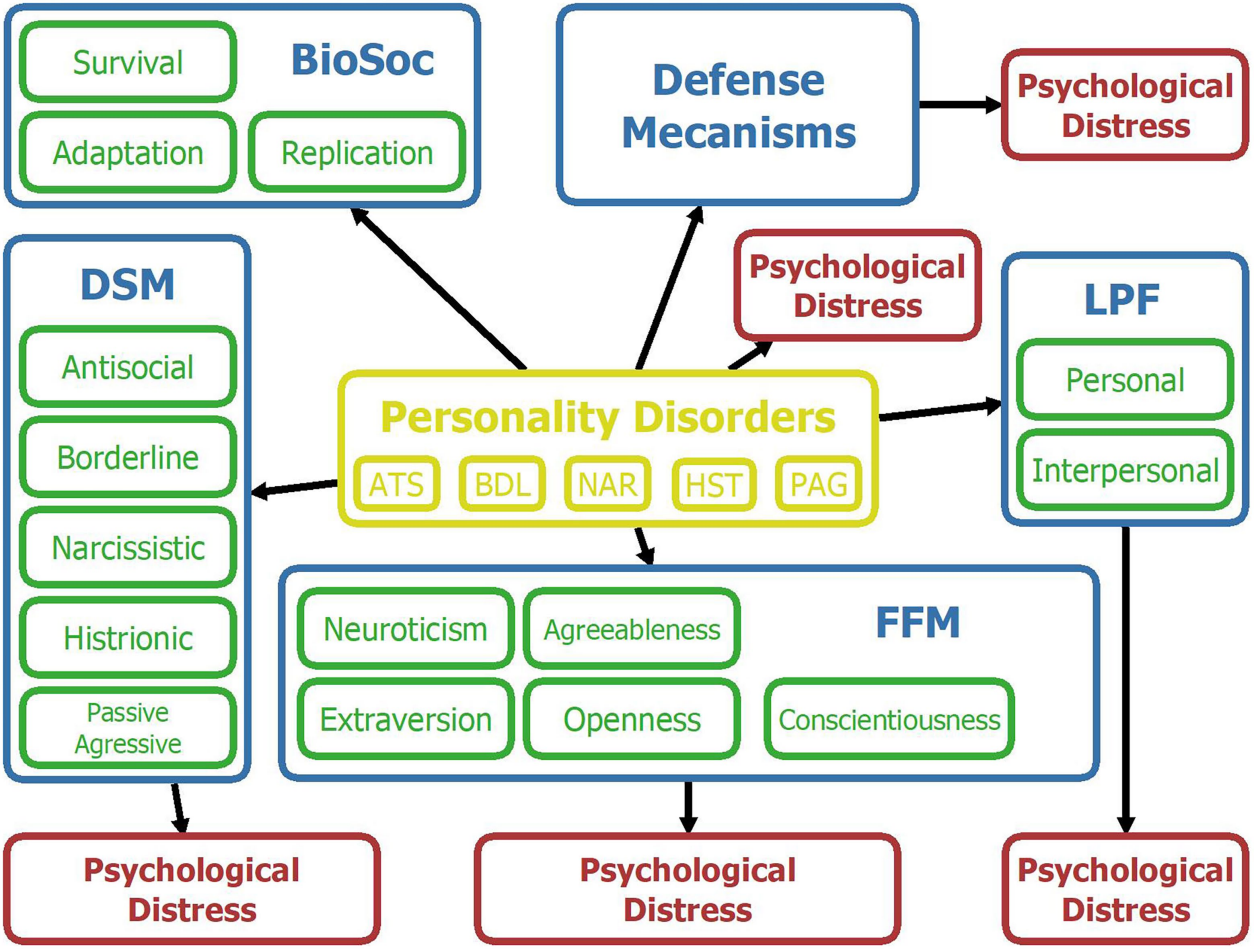
Map of variables for the Bayesian network. Yellow = Personality disorders; Blue = Psychological framework; Green = upper-level psychological constructs of a given framework; Red = Psychological distress. ATS = antisocial; BDL = borderline; NAR = narcissistic; HST = histrionic; PAG = passive-aggressive; BioSoc = Biosocial; DSM = Diagnostic and Statistical Manual of mental disorders; FFM = Five-Factor Model; LPF = Level of Personality Functioning.

**Figure 3:**
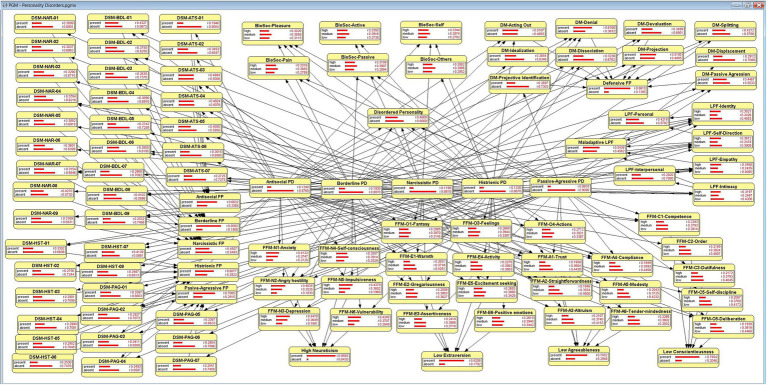
The Bayesian network, in OpenMarkov’s inference mode.

Figure 2 presents a schematic overview of the variables and relations included in the model, and Figure 3 shows a screenshot of the model described above before entering any finding in OpenMarkov’s inference mode. In addition, we include a map of the model’s variables to facilitate its understanding. However, given its length, it is published as supplementary material.

A working model stored in the format of OpenMarkov or Genie will be supplied upon request.

### Likelihood ratio for the improvement of diagnostic efficiency

From the probabilities elicited using knowledge engineering techniques, we have not only been able to obtain the conditional probability tables for the model but also very relevant information on the ranking and relative importance of each symptom with respect to the personality disorders studied.

Through the likelihood ratio, we can identify those symptoms that can most efficiently confirm or rule out the presence of personality disorders.

Tables 7 and 8 show the symptoms that have a positive likelihood ratio greater than 5 or a negative likelihood ratio smaller than 0.2 respectively, which will cause a moderate change in the post-test probabilities with respect to the pre-test probabilities.

**Table 7:** Symptoms having a positive likelihood ratio (given in parenthesis) higher or equal than 5 for some personality disorder.

ATS	BDL	NAR	HST	PAG
DSM - ATS 07 (7.06)	DSM - BDL 03 (7.75)	DSM - ATS 07 (6.44)	LPF - Intimacy (5.89)	LPF - Intimacy (5.39)
DSM - ATS 01 (6.69)	DSM - BDL 09 (6.36)		DSM - HST 06 (5.59)	LPF - Empathy (5.24)
LPF Intimacy (6.22)	LPF - Intimacy (6.17)		DSM - HST 01 (5.35)	
LPF Empathy (5.67)	LPF - Identity (5.86)		LPF - Identity (5.19)	
	LPF - Empathy (5.05)			
	DSM - BDL 04 (5.00)			
	DSM - BDL 07 (5.00)			

ATS = antisocial; BDL = borderline; NAR = narcissistic; HST = histrionic; PAG = passive-aggressive.

**Table 8:** Symptoms having a positive likelihood ratio (given in parenthesis) lower or equal than 0.2 for some personality disorder.

ATS	BDL	NAR	HST	PAG
LPF Empathy (0.18)	DSM - BDL 03 (0.13)	DSM - NAR 03 (0.11)	DSM - HST 06 (0.14)	MD - Passive-aggressive (0.15)
FFM Compliance (0.19)	LPF - Identity (0.14)	DSM - NAR 04 (0.13)	DSM - HST 01 (0.15)	DSM - PAG 07 (0.17)
MD - Acting out (0.20)	MD - Splitting (0.16)	DSM - NAR 09 (0.18)		
	MD - Devaluation (0.18)	DSM - NAR 02 (0.19)		
	DSM - BDL 02 (0.17)	DSM - NAR 01 (0.19)		
	DSM - BDL 04 (0.17)			

ATS = antisocial; BDL = borderline; NAR = narcissistic; HST = histrionic; PAG = passive-aggressive.

### Probing the model for content validity: Sensitivity analysis and strength of influence

Except for the graphical representation of the structure of the model or its usefulness in a practical application, it is difficult to ascertain the validity of the model by merely studying the parameters.

One way to solve this problem is by studying the strength influence for the links and the sensitivity analysis of the nodes. This allows us to assess the correctness of the conditional probability tables.

**Figure 4:**
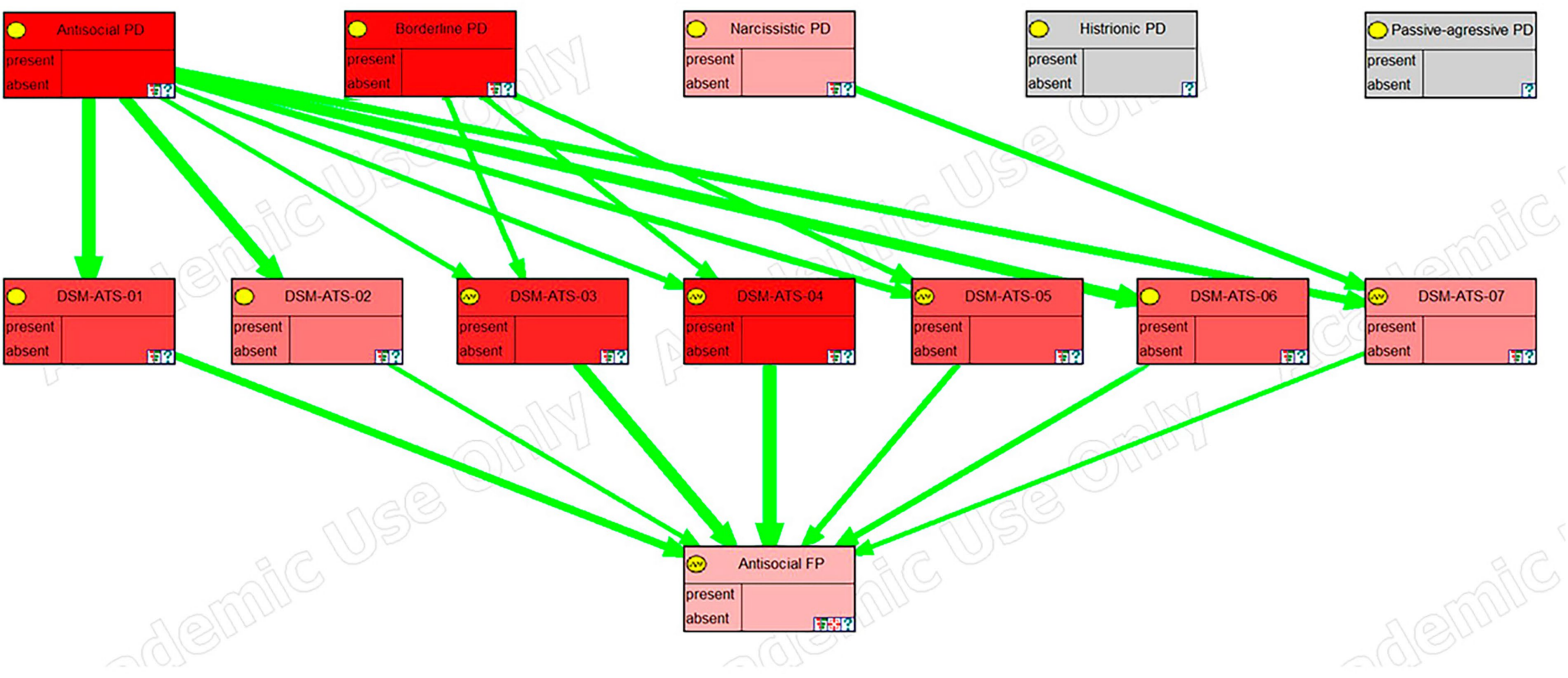
Sensitivity analysis, in GeNIE, for antisocial DSM symptoms.

**Figure 5:**
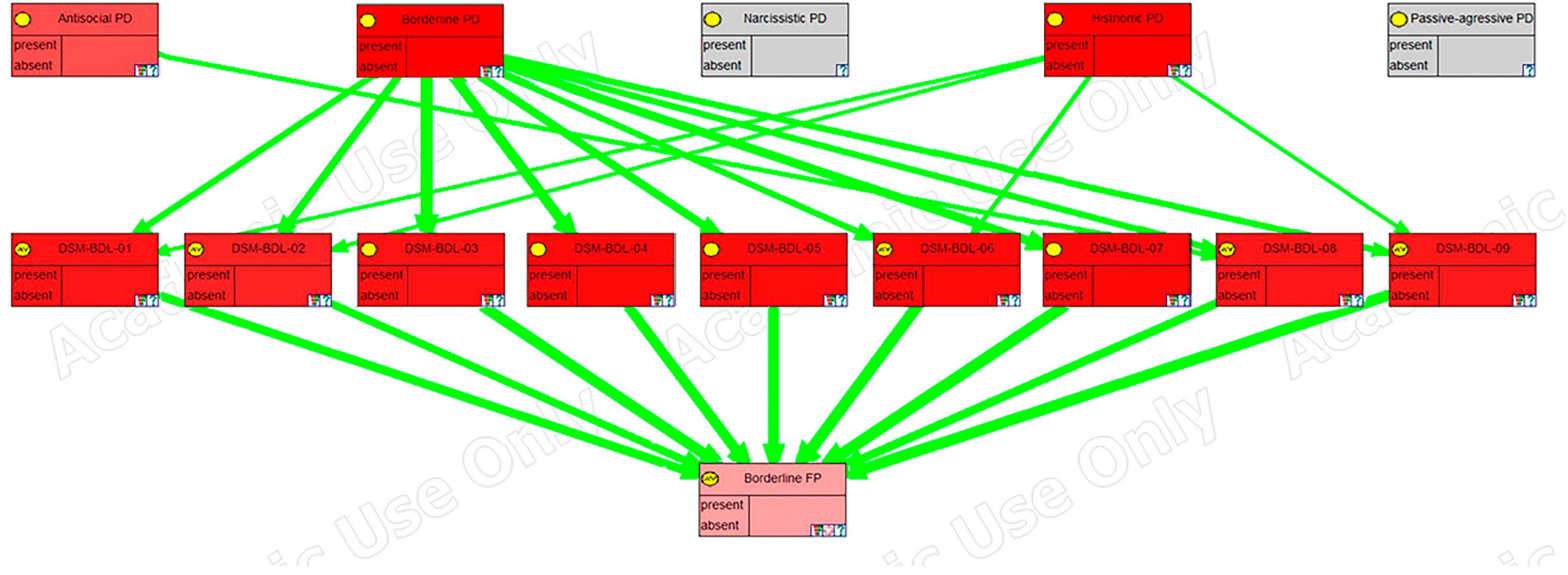
Sensitivity analysis, in GeNIE, for borderline DSM symptoms.

**Figure 6:**
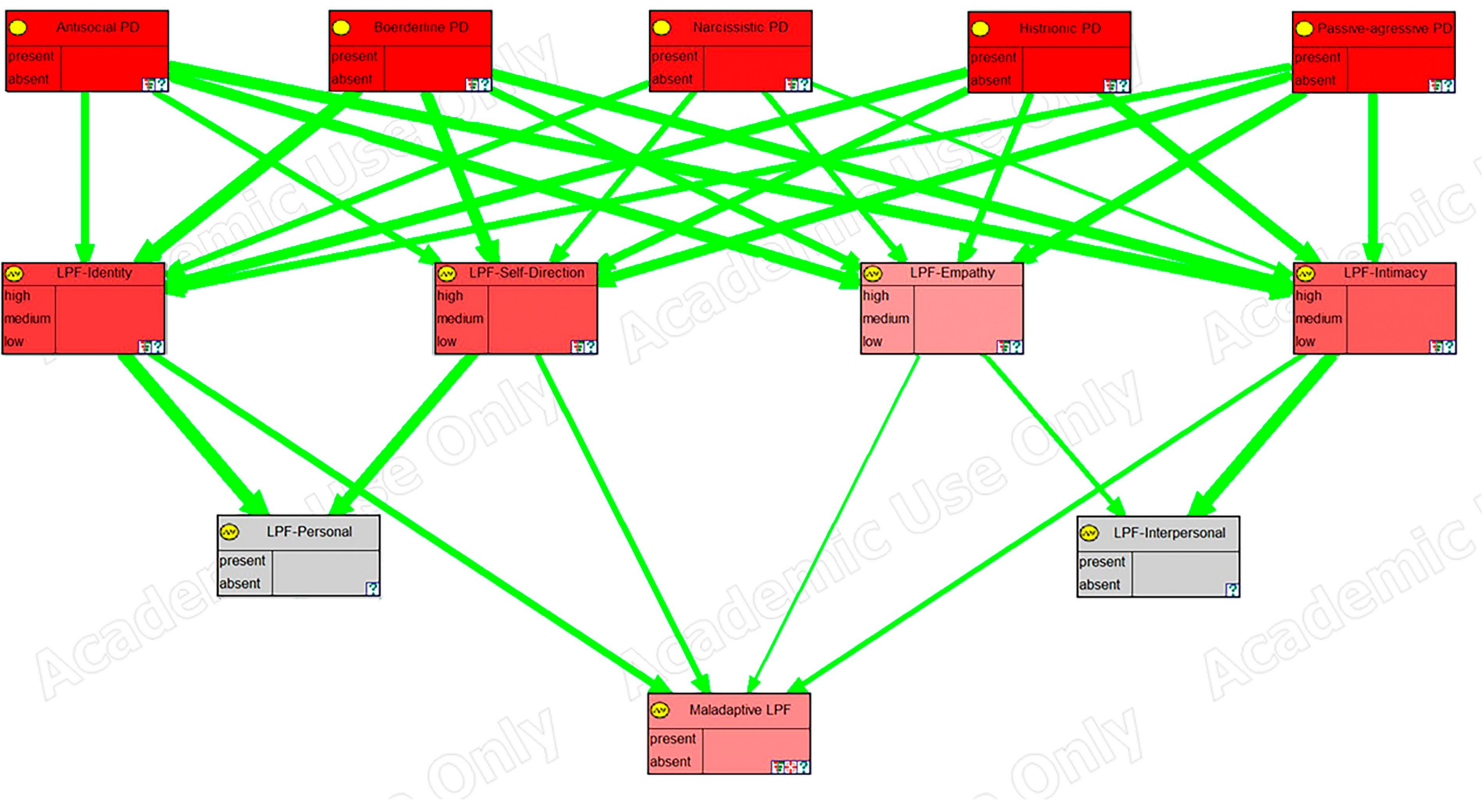
Sensitivity analysis, in GeNIE, for level of personality functioning scales.

The model has been exported from OpenMarkov to the academic version of GeNIE (Druzdzel, 1999) to take advantage of its graphing capabilities. In Figures 4–6, we can see a sensitivity analysis and the strength of influence for, respectively, the DSM antisocial symptoms, the DSM borderline symptoms, and the LPF scales.

In these images, the nodes in the top row correspond to the five personality disorders, the next row corresponds to the symptoms, traits, or scales of the framework, and the last row (the last two rows in the case of the last figure), corresponds to the node (s) representing psychological distress. Their color indicates the degree of sensitivity: the more redness, the higher the sensitivity.

Furthermore, green arrows indicate a direct influence, while red arrows would imply an inverse one. The thickness of the arrows shows the strength of the influence.

## Discussion

The purpose of this research is, through the incorporation of artificial intelligence techniques, to contribute to the improvement in the evaluation and treatment of personality disorders. These disorders, given their high prevalence and negative impact on all involved, require significant attention, especially considering the limitations that traditional methods have in assessing them.

To the best of our knowledge, no study has been conducted that includes the integration of a broad set of psychological variables useful for the evaluation of personality disorders of the dramatic and emotional type in a single model. Nor are there, to date, studies that combine for this purpose expert knowledge, bibliographical research, and statistical methods to integrate the different frameworks related to personality disorders.

To get these results we built a probabilistic graphical model using an open-source software, OpenMarkov (Arias et al., 2011). We obtained from the scientific literature and a group of experts following a Delphi method approach (Hasson et al., 2000; Waggoner et al., 2016). This model represents the relations between a broad set of psychological symptoms and the personality disorders of the dramatic and emotional cluster.

This model facilitates the assessment of personality disorders under a wide range of symptoms from different psychological frameworks. Additionally, with the probabilities obtained through the Delphi method, it has been possible to identify those psychological constructs with the highest diagnostic power for the confirmation or screening of personality disorders.

With respect to the model and its structure, the changes proposed by the experts regarding the relations found in the literature were minimal and, in any case, the changes were to introduce previously absent relations.

The fact that the relations initially included in the model, obtained from the literature, were hardly questioned gives confidence in the correctness of the model. Nevertheless, a bias or carry-over effect should not be ruled out, since the questionnaire specified those relations obtained from the scientific literature. Furthermore, the experts did not propose other psychological variables for inclusion in the model which is a positive indicator that the probabilistic graphical model is exhaustive in terms of the constructs or psychological variables.

Once the structure of the model was defined, the conditional probability tables were obtained from experts by the Delphi method showing that the average degree of agreement between the first and second rounds only increased by around 8%. This modest increase, which would hardly justify an additional Delphi round, occurs mainly because the standard deviation decreases as the scores get closer to the mean, so that, if we keep the same procedure as in the first round, reaching a higher consensus becomes more difficult even though, paradoxically, the results are closer to the mean. This finding is in line with the studies of Hasson et al. (2000) and Woudenberg (1991).

Furthermore, the percentage of items that were modified between the first and second rounds was considerable (≈80%), which seems to indicate a tendency to conform to the mean, probably due to peer pressure.

Given the conditional probabilities obtained for the model, we have been able to determine those symptoms that best allow us to confirm a suspected personality disorder in the clinical population and to rule out its presence in the general population. By identifying the symptoms with a higher positive likelihood ratio, we can develop a reduced measurement instrument to confirm the presence of personality disorders of the dramatic and emotional type in clinical settings. Conversely, by identifying symptoms with a lower negative likelihood ratio we can design a screening instrument to rule out the presence of personality disorders of the dramatic and emotional type in the general population. This would reduce the time needed between an initial consultation, where the patient’s clinical history is explored, and the moment of providing the treatment. Furthermore, the creation of a screening tool would allow us to reach more population and provide better access to mental health care without incurring the excessive cost of an indiscriminate complete psychological study.

The advantage of this approach with respect to the traditional method, in which the questionnaires used only include constructs from a single framework, is that, by using a questionnaire that explores the psychological constructs with the greatest likelihood ratio from different frameworks, we obtain a measurement instrument that, with the same extension, has greater diagnostic power (Grimes and Schulz, 2005).

The list of symptoms obtained in this study is quite short, so the presence or absence of these symptoms can be determined either by a questionnaire or by a directed interview in a short time. A common cut-off point in the literature has been used, namely LR+≥5 and LR−≤0.2. However, by modifying these cut-off points we can increase or reduce the number of symptoms, which will always be the most relevant, to tailor the desired length of the measurement instrument or the interview.

The most obvious aspect of this list of symptoms is the predominance of those from the DSM model. This was to be expected, since personality disorders are constructs defined on the basis of their symptoms; however, not all symptoms have the same diagnostic power, so this list is useful to rule out those that are either more common in the general population or less common in the clinical population, and can therefore be relegated to a second tier, with minimal loss of diagnostic power.

Other overrepresented symptoms in these lists are the level of personal functioning scales, which are present in the list for all personality disorders except for narcissistic personality disorder, evidence that it is, arguably, the least maladaptive personality disorder of the dramatic and emotional type.

Regarding the defense mechanisms, they appeared only among the symptoms with the lowest negative likelihood ratios. This could be because, although they are highly characteristic of personality disordered individuals, it is not uncommon to find them in the general population, so they are more useful to rule out the disorder than to confirm it. However, given the egosyntonic nature that personality disorders in this cluster tend to have, it is to be expected that coping mechanisms were in play to reduce the psychological distress caused by the effects of the disorder on the person’s life.

The five-factor model is hardly represented in the list of the most relevant symptoms for the same reason that defense mechanisms; the prevalence of high or low traits in the normal population is considerable. This supports the study of Rottman et al. (2010) that study that the five-factor model may not be sufficient to diagnose personality disorders. However, one possible solution would be to raise the cut-off points so that, by only considering the variables with the highest (or lowest) and most maladaptive scores as traits present, the prevalence in the normal population would be lowered and the specificity of these traits would be increased. Something similar occurs with Millon’s biosocial model whose polarities do not even appear in the list.

Although the model has not yet been validated with a representative sample of patients with personality disorders, the model shows good content validity, as it replicates the findings obtained in other studies using a different methodology. To illustrate this, we performed a sensitivity analysis on some variables of the model using the GeNIE software.

The sensitivity analysis for Antisocial DSM symptoms (Figure 4) showed how the 7 symptoms of this disorder relate primarily to antisocial personality disorder but also, in almost equal measure, to borderline personality disorder despite relating only through 3 of the 7 symptoms. Holthausen and Habel (2018) argued that borderline and antisocial personality disorders are two sides of the same coin and that they have a common underlying factor. They also claimed that the differences between the two disorders come from the way the symptoms manifest and not because of qualitative differences between the disorders. That is the reason why in the graph we see that the symptoms are related to both disorders in almost the same magnitude (depicted by the same intensity of red color).

Likewise, a sensitivity analysis for Borderline DSM symptoms shows its relation with the borderline personality disorder, but also, as mentioned in the previous paragraph, to antisocial personality disorder. However, we can also see that there is an even stronger relation with the histrionic personality disorder. Westen and Shedler (1999b), in one of their studies, make another classification of the disorders using a different methodology from the DSM. They suggest that some of the cases of borderline personality disorder would be better classified as histrionic personality disorder and in a new category called “emotional dysregulation.” Therefore, they propose a new category with symptoms taken from both. These findings are congruent with the graph shown in Figure 5.

A sensitivity analysis corresponding to the psychological variables of the level of personal functioning was also depicted (Figure 6). Sharp et al. (2015) proposed that there is a general factor “g” common to all personality disorders and a specific factor “s” that establishes the differences between the different personality disorders. Our sensitivity analysis showed how the level of personal functioning, measured by its four variables (identity, empathy, intimacy, and self-direction), was affected almost equally by all personality disorders, confirming that we were indeed measuring the “g” factor. However, it also showed how, for the clinically significant psychological distress that this “g” factor produces, the empathy construct had a significantly lower weight. This could be because although empathy is considered a positive attribute, in certain environments, such as finance and politics, is not very adaptive. That is, a lack of empathy is useful to thrive; at the very least, it may not be seen as dysfunctional as the lack of any of the other constructs. This is congruent with some previous work on empathy (Olson, 2012).

The Bayesian network developed in this research has different applications, we will focus on just three.

First, the principal application of a Bayesian network is to compute the posterior probabilities of the states of the variables given a set of findings. In our context, this allows us to determine the probability of each personality disorders given the patient’s symptoms. The probability score should not, necessarily, be interpreted in absolute terms, but in relation to the score obtained in the other personality disorders, taking into account that if the x-axis represented the weighted number of symptoms present and the y-axis the probabilities, the function would have a sigmoid shape.

While a therapist is necessary for both the determination of the symptoms and the interpretation of the results, the system can interactively guide the psychological assessment, saving time and facilitating a comprehensive exploration of all the related psychological variables. An advantage with respect to the traditional diagnostic method is the possibility of making a more complete examination, while reducing the evaluator’s biases. Although the use of a new tool may initially require an additional effort, this is rewarded with a reduction in the time for the personal interview by being able to directly address the most relevant aspects of the patient’s narrative.

The assessment offered by the system is based on the probabilities of both the presence of personality disorders and the likelihood that the evaluated symptoms produce clinically significant psychological distress. The therapist can decide whether to assess all the psychological variables in the model for greater accuracy or to assess a reduced set, in which case the system takes a probabilistic value for the variables whose status is unknown based on the conditional probability tables and the findings entered in the adjacent nodes.

The second application of the system is the possibility of performing a sensitivity analysis—, once the findings have been introduced and an assessment has been obtained,—to determine which symptoms contribute most to the diagnosis. These symptoms constitute the therapeutic targets that may optimize the treatment to reduce the psychological distress as efficiently as possible. However, the fact that a psychological variable has the greatest contribution to the diagnosis does not mean that it is the easiest to be treated, so sensitivity analysis should be regarded as an additional aid to the therapist rather than a straightforward guide.

The third application is the use of the model as an educational tool for psychologists in training. Since there is the possibility of updating, in real-time, a diagnosis based on the symptoms of a patient’s psychological profile, a student can see how the diagnosis changes when including or excluding certain symptoms. This, combined with a comprehensive listing of related variables, text boxes with detailed information about symptoms and their characteristics, and color coding of the scores to determine whether the change is positive or negative, we have a simulation tool with great potential to complement other more traditional training methods.

It can be argued that some of the decisions made for the modeling could be somewhat arbitrary, such as the discretization of nodes, the choice of canonical models, or their parameters. However, even the simplest Bayesian networks (i.e., the naive Bayes) are very robust to both imprecise data and approximate assumptions. One of the reasons for such good performance is that, when faced with classification tasks, absolute probabilities between nodes in the model are not as important as the relative probabilities and ranking; that is, if the state of one node is more probable than another, this is be reflected in the model through the probabilities, even if these are not exact (Rish, 2001; Zhang, 2005). This property is maintained with the parameters and the methodology used.

However, one of the next steps to address some of the limitations of this study is to refine the model with statistical data obtained empirically as soon as it is available. Although this statistical data would not be without bias either, it would allow us to fit the model to different populations for a more accurate diagnosis.

Furthermore, in the near future, we will validate the model in a clinical setting to determine its suitability for the assessment and treatment of personality disorders of the dramatic and emotional type. Similarly, it will be of interest to explore the applicability of the model in the training of new psychologists.

Other lines of work aimed at improving the diagnosis and treatment of personality disorders would be taking into account other factors such as ease of treatment and the expectations of success. In this sense, part of the work has already been done by using the Delphi method to measure the psychological distress that each symptom can produce.

The use of artificial intelligence techniques in the field of psychology is an innovative approach that complements traditional techniques used for the investigation and assessment of psychological disorders. Although in this research we have focused on a subset of personality disorders, the methodology is applicable not only to the rest of personality disorders, but also to other psychological conditions whose causality is multifactorial and where empirical data is scarce.

## Data availability statement

The raw data supporting the conclusions of this article will be made available by the authors, without undue reservation.

## Author contributions

JG-Fs contribution was in the analysis of the data and the modeling of the Bayesian network. FDs contribution was in the area of artificial intelligence, Bayesian networks, and canonical models. MCs contribution was in the area of psychology, diagnosis and treatment of personality disorders, and data gathering from the group of experts. All authors contributed to the article and approved the submitted version.

## Conflict of interest

The authors declare that the research was conducted in the absence of any commercial or financial relationships that could be construed as a potential conflict of interest.

## Publisher’s note

All claims expressed in this article are solely those of the authors and do not necessarily represent those of their affiliated organizations, or those of the publisher, the editors and the reviewers. Any product that may be evaluated in this article, or claim that may be made by its manufacturer, is not guaranteed or endorsed by the publisher.
