# Supplementary material for: Probabilistic graphical model for the evaluation of the emotional and dramatic personality disorders
Source: Front Psychol. 2022 Nov 25;13:996609. doi: 10.3389/fpsyg.2022.996609 (PMC9732555; doi:10.3389/fpsyg.2022.996609)
Supplement: Supplementary file 1 [file Data_Sheet_1.PDF]

Supplementary Material

1 Supplementary Data

We provide a map of variables for the Bayesian network to allow for a better understanding of the model. We have based it on figures 1 and 2 of the manuscript to include all the nodes of the model dividing them into three tables.

The nodes described in Table 1 correspond to the first level of the BN2O model pertaining to the personality disorders; they are drawn in yellow in figure 2. These nodes are "variables of interest", i.e., they are nodes for which we want to find out the probability that they are in one state or another.

These variables do not have ancestor nodes, so we use the prevalence of these disorders in the clinical population to determine the prior probabilities.

The nodes in Table 2 correspond to the psychological features that a person may exhibit that help us determine whether a personality disorder is present. Regarding figure 1 of the manuscript, these nodes would be at the lower level of the BN2O model and are drawn in blue in figure 2.

The symptoms´ ancestor nodes are the variables representing the personality disorders described in Table 1. The successor nodes are the variables representing the psychological distress we shall see below in Table 3.

The nodes presented in Table 3 represent the psychological distress that a person may experience when a given combination of symptoms are present. Therefore, the ancestor nodes are those symptoms capable of generating psychological distress. These variables have no successor nodes.

These nodes correspond to the augmented level of the BN2O model in figure 1 and are drawn in red in figure 2.

2 Supplementary Tables

Table 1

First level of the BN2O model – personality disorders.

Variable	Description	States	Ancestor nodes
Antisocial PD	Antisocial personality disorder	present/absent	--
Borderline PD	Borderline personality disorder	present/absent	--
Narcissistic PD	Narcissistic personality disorder	present/absent	--
Histrionic PD	Histrionic personality disorder	present/absent	--
Passive aggressive PD	Passive aggressive personality disorder	present/absent	--

Table 2

Second level of the BN2O model – Symptoms.

Variable	Description	States	Ancestor nodes
DSM-ATS-01	1st symptom of antisocial personality disorder as described in the DSM.	present/<u>absent</u>	Antisocial PD
DSM-ATS-02	2nd symptom of antisocial personality disorder as described in the DSM.	present/<u>absent</u>	Antisocial PD
DSM-ATS-03	3rd symptom of antisocial personality disorder as described in the DSM.	present/<u>absent</u>	Antisocial PD<br>Borderline PD
DSM-ATS-04	4th symptom of antisocial personality disorder as described in the DSM.	present/<u>absent</u>	Antisocial PD<br>Borderline PD
DSM-ATS-05	5th symptom of antisocial personality disorder as described in the DSM.	present/<u>absent</u>	Antisocial PD<br>Borderline PD
DSM-ATS-06	6th symptom of antisocial personality disorder as described in the DSM.	present/<u>absent</u>	Antisocial PD
DSM-ATS-07	7th symptom of antisocial personality disorder as described in the DSM.	present/<u>absent</u>	Antisocial PD<br>Narcissistic PD

Variable	Description	States	Ancestor nodes
DSM-BDL-01	1st symptom of borderline personality disorder as described in the DSM.	present/absent	Borderline PD<br>Histrionic PD
DSM-BDL-02	2nd symptom of borderline personality disorder as described in the DSM.	present/absent	Borderline PD<br>Histrionic PD
DSM-BDL-03	3rd symptom of borderline personality disorder as described in the DSM.	present/absent	Borderline PD
DSM-BDL-04	4th symptom of borderline personality disorder as described in the DSM.	present/absent	Borderline PD
DSM-BDL-05	5th symptom of borderline personality disorder as described in the DSM.	present/absent	Borderline PD
DSM-BDL-06	6th symptom of borderline personality disorder as described in the DSM.	present/absent	Borderline PD<br>Histrionic PD
DSM-BDL-07	7th symptom of borderline personality disorder as described in the DSM.	present/absent	Borderline PD
DSM-BDL-08	8th symptom of borderline personality disorder as described in the DSM.	present/absent	Borderline PD<br>Antisocial PD
DSM-BDL-09	9th symptom of borderline personality disorder as described in the DSM.	present/absent	Borderline PD<br>Histrionic PD
DSM-NAR-01	1st symptom of narcissistic personality disorder as described in the DSM.	present/absent	Narcissistic PD
DSM-NAR-02	2nd symptom of narcissistic personality disorder as described in the DSM.	present/absent	Narcissistic PD
DSM-NAR-03	3rd symptom of narcissistic personality disorder as described in the DSM.	present/absent	Narcissistic PD
DSM-NAR-04	4th symptom of narcissistic personality disorder as described in the DSM.	present/absent	Narcissistic PD<br>Histrionic PD
DSM-NAR-05	5th symptom of narcissistic personality disorder as described in the DSM.	present/absent	Narcissistic PD

Variable	Description	States	Ancestor nodes
DSM-NAR-06	6th symptom of narcissistic personality disorder as described in the DSM.	present/absent	Narcissistic PD
DSM-NAR-07	7th symptom of narcissistic personality disorder as described in the DSM.	present/absent	Narcissistic PD<br>Antisocial PD
DSM-NAR-08	8th symptom of narcissistic personality disorder as described in the DSM.	present/absent	Narcissistic PD<br>Passive aggressive PD
DSM-NAR-09	9th symptom of narcissistic personality disorder as described in the DSM.	present/absent	Narcissistic PD
DSM-HST-01	1st symptom of histrionic personality disorder as described in the DSM.	present/absent	Histrionic PD<br>Narcissistic PD
DSM-HST-02	2nd symptom of histrionic personality disorder as described in the DSM.	present/absent	Histrionic PD
DSM-HST-03	3rd symptom of histrionic personality disorder as described in the DSM.	present/absent	Histrionic PD
DSM-HST-04	4th symptom of histrionic personality disorder as described in the DSM.	present/absent	Histrionic PD
DSM-HST-05	5th symptom of histrionic personality disorder as described in the DSM.	present/absent	Histrionic PD
DSM-HST-06	6th symptom of histrionic personality disorder as described in the DSM.	present/absent	Histrionic PD
DSM-HST-07	7th symptom of histrionic personality disorder as described in the DSM.	present/absent	Histrionic PD<br>Borderline PD
DSM-HST-08	8th symptom of histrionic personality disorder as described in the DSM.	present/absent	Histrionic PD<br>Borderline PD
DSM-PAG-01	1st symptom of passive-aggressive personality disorder as described in the DSM.	present/absent	Passive aggressive PD<br>Antisocial PD

Variable	Description	States	Ancestor nodes
DSM-PAG-02	2nd symptom of passive-aggressive personality disorder as described in the DSM.	present/absent	Passive aggressive PD<br>Histrionic PD
DSM-PAG-03	3rd symptom of passive-aggressive personality disorder as described in the DSM.	present/absent	Passive aggressive PD<br>Antisocial PD
DSM-PAG-04	4th symptom of passive-aggressive personality disorder as described in the DSM.	present/absent	Passive aggressive PD<br>Antisocial PD
DSM-PAG-05	5th symptom of passive-aggressive personality disorder as described in the DSM.	present/absent	Passive aggressive PD<br>Narcissistic PD
DSM-PAG-06	6th symptom of passive-aggressive personality disorder as described in the DSM.	present/absent	Passive aggressive PD
DSM-PAG-07	7th symptom of passive-aggressive personality disorder as described in the DSM.	present/absent	Passive aggressive PD
FFM-N1-Anxiety	Anxiety trait from the FFM neuroticism domain.	high/medium/low	Antisocial PD<br>Borderline PD
FFM-N2-Angry hostility	Hostility trait from the FFM neuroticism domain.	high/medium/low	Antisocial PD<br>Borderline PD<br>Narcissistic PD<br>Passive aggressive PD
FFM-N3-Depression	Depression trait from the FFM neuroticism domain.	high/medium/low	Borderline PD<br>Histrionic PD
FFM-N4-Self-consciousness	Self-consciousness trait from the FFM neuroticism domain.	high/medium/low	Antisocial PD
FFM-N5-Impulsiveness	Impulsiveness trait from the FFM neuroticism domain.	high/medium/low	Antisocial PD<br>Borderline PD
FFM-N6-Vulnerability	Vulnerability trait from the FFM neuroticism domain.	high/medium/low	Borderline PD<br>Histrionic PD

Variable	Description	States	Ancestor nodes
FFM-E1-Warmth	Warmth trait from the FFM extraversion domain.	high/medium/low	Antisocial PD<br>Borderline PD<br>Narcissistic PD
FFM-E2-Gregariousness	Gregariousness trait from the FFM extraversion domain.	high/medium/low	Antisocial PD<br>Borderline PD<br>Histrionic PD
FFM-E3-Assertiveness	Assertiveness trait from the FFM extraversion domain.	high/medium/low	Narcissistic PD<br>Passive aggressive PD
FFM-E4-Activity	Activity trait from the FFM extraversion domain.	high/medium/low	Histrionic PD
FFM-E5-Excitement seeking	Excitement seeking trait from the FFM extraversion domain.	high/medium/low	Antisocial PD<br>Narcissistic PD<br>Histrionic PD
FFM-E6-Positive emotions	Positive emotions trait from the FFM extraversion domain.	high/medium/low	Histrionic PD
FFM-O1-Fantasy	Fantasy trait from the FFM openness domain.	high/medium/low	Borderline PD<br>Narcissistic PD<br>Histrionic PD
FFM-O3-Feelings	Feelings trait from the FFM openness domain.	high/medium/low	Histrionic PD
FFM-O4-Actions	Actions trait from the FFM openness domain.	high/medium/low	Borderline PD<br>Histrionic PD
FFM-A1-Trust	Trust trait from the FFM agreeableness domain.	high/medium/low	Antisocial PD<br>Borderline PD<br>Narcissistic PD<br>Histrionic PD<br>Passive aggressive PD
FFM-A2-Straightforwardness	Straightforwardness trait from the FFM agreeableness domain.	high/medium/low	Antisocial PD<br>Borderline PD<br>Narcissistic PD<br>Passive aggressive PD

Variable	Description	States	Ancestor nodes
FFM-A3-Altruism	Altruism trait from the FFM agreeableness domain.	high/medium/low	Antisocial PD<br>Narcissistic PD
FFM-A4-Compliance	Compliance trait from the FFM agreeableness domain.	high/medium/low	Antisocial PD<br>Borderline PD<br>Narcissistic PD<br>Passive aggressive PD
FFM-A5-Modesty	Modesty trait from the FFM agreeableness domain.	high/medium/low	Antisocial PD<br>Narcissistic PD
FFM-A6-Tender-mindedness	Tender-mindedness trait from the FFM agreeableness domain.	high/medium/low	Antisocial PD<br>Narcissistic PD
FFM-C1-Competence	Competence trait from the FFM conscientiousness domain.	high/medium/low	Borderline PD<br>Narcissistic PD<br>Passive aggressive PD
FFM-C2-Order	Order trait from the FFM conscientiousness domain.	high/medium/low	Borderline PD
FFM-C3-Dutifulness	Dutifulness trait from the FFM conscientiousness domain.	high/medium/low	Antisocial PD<br>Passive aggressive PD
FFM-C5-Self-discipline	Self-discipline trait from the FFM conscientiousness domain.	high/medium/low	Antisocial PD<br>Passive aggressive PD
FFM-C6-Deliberation	Deliberation trait from the FFM conscientiousness domain.	high/medium/low	Antisocial PD<br>Borderline PD<br>Histrionic PD
LPF-Identity	Identity scale from the level of personality functioning as described in the general criteria of the DSM-5.	high/medium/low	Antisocial PD<br>Borderline PD<br>Narcissistic PD<br>Histrionic PD<br>Passive aggressive PD
LPF-Self-Direction	Self-Direction scale from the level of personality functioning as described in the general criteria of the DSM-5.	high/medium/low	Antisocial PD<br>Borderline PD<br>Narcissistic PD<br>Histrionic PD<br>Passive aggressive PD

Variable	Description	States	Ancestor nodes
LPF-Empathy	Empathy scale from the level of personality functioning as described in the general criteria of the DSM-5.	high/medium/low	Antisocial PD<br>Borderline PD<br>Narcissistic PD<br>Histrionic PD<br>Passive aggressive PD
LPF-Intimacy	Intimacy scale from the level of personality functioning as described in the general criteria of the DSM-5.	high/medium/low	Antisocial PD<br>Borderline PD<br>Narcissistic PD<br>Histrionic PD<br>Passive aggressive PD
DM-Acting Out	Acting out defense mechanism as described in the annex B of the DSM-IV-TR.	present/absent	Antisocial PD<br>Borderline PD<br>Histrionic PD
DM-Idealization	Idealization defense mechanism as described in the annex B of the DSM-IV-TR.	present/absent	Borderline PD
DM-Projective Identification	Projective identification defense mechanism as described in the annex B of the DSM-IV-TR.	present/absent	Passive aggressive PD
DM-Denial	Denial defense mechanism as described in the annex B of the DSM-IV-TR.	present/absent	Antisocial PD<br>Borderline PD<br>Narcissistic PD<br>Histrionic PD
DM-Dissociation	Dissociation defense mechanism as described in the annex B of the DSM-IV-TR.	present/absent	Antisocial PD<br>Narcissistic PD<br>Histrionic PD
DM-Devaluation	Devaluation defense mechanism as described in the annex B of the DSM-IV-TR.	present/absent	Borderline PD<br>Narcissistic PD
DM-Projection	Projection defense mechanism as described in the annex B of the DSM-IV-TR.	present/absent	Antisocial PD<br>Narcissistic PD
DM-Splitting	Splitting defense mechanism as described in the annex B of the DSM-IV-TR.	present/absent	Borderline PD<br>Histrionic PD

Variable	Description	States	Ancestor nodes
DM-Displacement	Displacement defense mechanism as described in the annex B of the DSM-IV-TR.	present/absent	Passive aggressive PD
DM-Passive Aggression	Passive aggression defense mechanism as described in the annex B of the DSM-IV-TR.	present/absent	Borderline PD<br>Histrionic PD<br>Passive aggressive PD
BioSoc-Pleasure	“Pleasure” evolutionary task from Millon´s Biosocial model.	high/medium/low	Borderline PD<br>Narcissistic PD<br>Histrionic PD<br>Passive aggressive PD
BioSoc-Pain	“Pain” evolutionary task from Millon´s Biosocial model.	high/medium/low	Borderline PD<br>Histrionic PD<br>Passive aggressive PD
BioSoc-Active	“Active” evolutionary task from Millon´s Biosocial model.	high/medium/low	Narcissistic PD<br>Histrionic PD
BioSoc-Passive	“Passive” evolutionary task from Millon´s Biosocial model.	high/medium/low	Borderline PD<br>Histrionic PD<br>Passive aggressive PD
BioSoc-Self	“Self” evolutionary task from Millon´s Biosocial model.	high/medium/low	Antisocial PD<br>Narcissistic PD<br>Histrionic PD
BioSoc-Others	“Others” evolutionary task from Millon´s Biosocial model.	high/medium/low	Histrionic PD

Note. Default state for the variable appears as underlined.

Table 3

Augmented level of the BN2O model – Psychological distress.

Variable	Description	States	Ancestor nodes
Disordered Personality	Psychological distress caused by a personality disorder.	present/absent	Antisocial PD<br>Borderline PD<br>Narcissistic PD<br>Histrionic PD<br>Passive aggressive PD
Antisocial FP	Psychological distress caused by an antisocial functioning pattern.	present/absent	DSM-ATS-01<br>DSM-ATS-02<br>DSM-ATS-03<br>DSM-ATS-04<br>DSM-ATS-05<br>DSM-ATS-06<br>DSM-ATS-07
Borderline FP	Psychological distress caused by a borderline functioning pattern.	present/absent	DSM-BDL-01<br>DSM-BDL-02<br>DSM-BDL-03<br>DSM-BDL-04<br>DSM-BDL-05<br>DSM-BDL-06<br>DSM-BDL-07<br>DSM-BDL-08<br>DSM-BDL-09
Narcissistic FP	Psychological distress caused by a narcissistic functioning pattern.	present/absent	DSM-NAR-01<br>DSM-NAR-02<br>DSM-NAR-03<br>DSM-NAR-04<br>DSM-NAR-05<br>DSM-NAR-06<br>DSM-NAR-07<br>DSM-NAR-08<br>DSM-NAR-09
Histrionic FP	Psychological distress caused by a histrionic functioning pattern.	present/absent	DSM-HST-01<br>DSM-HST-02<br>DSM-HST-03<br>DSM-HST-04<br>DSM-HST-05<br>DSM-HST-06<br>DSM-HST-07<br>DSM-HST-08

Variable	Description	States	Ancestor nodes
Passive-Aggressive FP	Psychological distress caused by a passive aggressive functioning pattern.	present/absent	DSM-PAG-01<br>DSM-PAG-02<br>DSM-PAG-03<br>DSM-PAG-04<br>DSM-PAG-05<br>DSM-PAG-06<br>DSM-PAG-07
High Neuroticism	Psychological distress caused by having a high neuroticism.	present/absent	FFM-N1-Anxiety<br>FFM-N2-Angry hostility<br>FFM-N3-Depression<br>FFM-N4-Self-consciousness<br>FFM-N5-Impulsiveness<br>FFM-N6-Vulnerability
Low Extraversion	Psychological distress caused by having a low extraversion.	present/absent	FFM-E1-Warmth<br>FFM-E2-Gregariousness<br>FFM-E3-Assertiveness<br>FFM-E4-Activity<br>FFM-E5-Excitement seeking<br>FFM-E6-Positive emotions
Low Agreeableness	Psychological distress caused by having a low agreeableness.	present/absent	FFM-A1-Trust<br>FFM-A2-Straightforwardness<br>FFM-A3-Altruism<br>FFM-A4-Compliance<br>FFM-A5-Modesty<br>FFM-A6-Tender-mindedness
Low Conscientiousness	Psychological distress caused by having a low conscientiousness.	present/absent	FFM-C1-Competence<br>FFM-C2-Order<br>FFM-C3-Dutifulness<br>FFM-C5-Self-discipline<br>FFM-C6-Deliberation
Maladaptive LPF	Psychological distress caused by having a maladaptive level of personality functioning.	present/absent	LPF-Identity<br>LPF-Self-Direction<br>LPF-Empathy<br>LPF-Intimacy
LPF-Personal	Psychological distress caused by having a maladaptive level of personality functioning taking only into account the personal aspects.	present/absent	LPF-Identity<br>LPF-Self-Direction

Variable	Description	States	Ancestor nodes
LPF-Interpersonal	Psychological distress caused by having a maladaptive level of personality functioning taking only into account the interpersonal aspects.	present/absent	LPF-Empathy<br>LPF-Intimacy
Defensive FP	Psychological distress caused by having an excess of immature copying mechanism.	present/absent	DM-Acting Out<br>DM-Idealization<br>DM-Projective Identification<br>DM-Denial<br>DM-Dissociation<br>DM-Devaluation<br>DM-Projection<br>DM-Splitting<br>DM-Displacement<br>DM-Passive Aggression
